# Lactate in the burn patient

**DOI:** 10.1186/cc14225

**Published:** 2015-03-16

**Authors:** EH Herrero, M Sánchez, L Cachafeiro, A Agrifoglio, B Galván, MJ Asensio, A García de Lorenzo

**Affiliations:** 1 Hospital La Paz, Madrid, Spain

## Introduction

Severe burns result in rapid loss of intravascular volume due to development of a severe capillary leak and hypovolemic shock. It is widely accepted that traditional markers, such as blood pressure and urinary output, are useful but do not sufficiently reflect global perfusion, regional microcirculation or reversal shock. Blood lactate concentration is widely used in ICUs as a reliable prognostic marker of global tissue hypoxia. Our aim is to determine whether the percentage of lactate clarified in the first 24 hours is valid as a guide for resuscitation.

## Methods

We prospectively studied 143 consecutive burn patients admitted to our Burn Unit. Sociodemographics and comorbidities data were recorded. Clinical data were collected to calculate the Acute Burn Severity Index. Resuscitation according to the Parkland formula was guided by a urinary output of 0.5 to 1 ml/hour and the results of monitoring the blood pressure. Crystalloid solution (Ringer´s acetate) was given exclusively during the first 24 hours. Early surgical excision of burn eschar and early coverage of excised burn wounds with autografts was performed. Initial and subsequent serum lactate levels were measured to calculate lactate clearance according to the formula: lactate basal - lactate at 24 hours / lactate basal × 100. The primary outcome was mortality.

## Results

During a period of 2 years we studied 143 patients; their mean age was 46.98 ± 19.38 years, mean TBSA burn injury of the study population was 22.82 ± 20.25. The flame was the most frequent mechanism. A total of 83 patients were in mechanical ventilation and 13.6% of them developed ARDS. The mortality range in the study group was 17%.

Serum lactate at admission ≥2 mmol/l was associated with 31.3% mortality versus 6% in patients with a serum lactate at admission <2 mmol/l (*P *< 0.05). Length of time to lactate normalization variable is associated with mortality (*P *< 0.02). The average lactate normalization time was 4.6 days in nonsurvivors while in survivors it was 2.02 days. A relation does not exist between the lactate clearance and mortality in all patients.

## Conclusion

The length of time to lactate normalization in the severe burn patient is a marker of survival and a simple parameter to guide the endpoint of resuscitation; however, the percentage of lactate clarified in the first 24 hours is not valid.

